# Dietary intervention with narrow-leaved cattail rhizome flour (*Typha angustifolia* L.) prevents intestinal inflammation in the trinitrobenzenesulphonic acid model of rat colitis

**DOI:** 10.1186/1472-6882-12-62

**Published:** 2012-05-04

**Authors:** Andréa Costa Fruet, Leonardo Noboru Seito, Vera Lúcia Mores Rall, Luiz Claudio Di Stasi

**Affiliations:** 1 Laboratory of Phytomedicines, Department of Pharmacology, Instituto de Biociências, São Paulo State University (UNESP), Botucatu, 18618-970SP, Brazil; 2 Department of Microbiology and Immunology, Instituto de Biociências, São Paulo State University (UNESP), Botucatu, 18618-970SP, Brazil

## Abstract

**Background:**

Inflammatory bowel disease (IBD) is a chronic inflammation of the intestinal epithelium that is driven by the intestinal immune system, oxidative stress and the loss of tolerance to the luminal microbiota. The use of dietary products containing ingredients such as fibres and carbohydrates and/or antioxidant compounds have been used as a therapeutic strategy for intestinal diseases because these products are considered effective in the modulation of the immune system and colonic microbiota. We investigated the beneficial effects of cattail rhizome flour (*Typha angustifolia* L.) in the trinitrobenzenesulphonic acid (TNBS) model of rat colitis. In addition, we investigated the effects of cattail rhizome flour on the intestinal anti-inflammatory activity of prednisolone, which is a reference drug that is used for treatment of human IBD.

**Methods:**

The present study included the preparation of flour from rhizomes of cattail (*Typha angustifolia* L.); an evaluation of the qualitative phytochemical profile of cattail rhizomes; an evaluation of the efficacy of cattail rhizome flour in TNBS-induced rat colitis; an evaluation of the synergistic effects of cattail rhizome flour on the intestinal anti-inflammatory activity of prednisolone; and macroscopic, clinical, biochemical, histopathological and microbiological studies to assess the healing effects of cattail rhizome flour and its synergistic effects in TNBS-induced rat colitis. The data were analysed by ANOVA, Kruskal-Wallis and χ^2^ tests.

**Results:**

We tested several concentrations of cattail rhizome flour and found that dietary supplementation with 10% cattail rhizome flour showed the best effects at reducing the extension of the lesion, the colon weight ratio, adherences to adjacent organs and diarrhoea. These effects were related to inhibition of myeloperoxidase (MPO) and alkaline phosphatase (AP) activities and an attenuation of glutathione (GSH) depletion. The 10% cattail rhizome flour was as effective as prednisolone, and no synergistic effects were observed. Saponins, flavonoids and coumarins were detected in the rhizome flour. No changes were observed in the total number of lactic bacteria after dietary supplementation with cattail rhizome flour.

**Conclusions:**

Dietary supplementation with 10% cattail rhizome flour and its combination with prednisolone prevent TNBS-induced colonic damage in rats, but no synergistic effects were observed. The prevention of TNBS-induced colon damage was associated with an improvement in intestinal oxidative stress, which likely resulted from the antioxidant properties of the active compounds detected in the cattail rhizome. This protective effect was not related to an improvement in lactic bacteria counts.

## Background

Inflammatory Bowel Disease (IBD) is a collective term for a group of chronic intestinal inflammation states of the small and/or large intestines that encompasses ulcerative colitis (UC), a chronic mucosal and sub-mucosal inflammation of the large intestine and rectum, and Crohn’s disease (CD), a chronic transmural inflammation of all/any part of the gastro-intestinal tract [1]. Although much progress has been made in understanding the pathogenesis of human IBD, its aetiology has not been defined [2]; however, development of tissue injury is attributed to immune system alterations, reactive oxygen species and the loss of normal tolerance to the host [3-5]. Interestingly, there is increasing experimental evidence to support a role for luminal bacteria in the initiation and development of the intestinal inflammatory process, and this is probably related to an imbalance in the intestinal microflora (i.e., a relative predominance of aggressive bacteria and an insufficient amount of protective species) [1,5,6].

Dietary components, primarily dietary fibre, have proven to be beneficial in maintaining remission in human and experimental ulcerative colitis, and the protective effect of fibre is related to an increase in the luminal production of short-chain fatty acids (SCFAs), including acetate, propionate and butyrate [7]. Butyrate has been reported to be an important factor in the maintenance of healthy function in colorectal mucosa and the major fuel source for colonocytes [8]. Several studies have suggested that some food products, including dietary fibre, germinated barley foodstuff, inulin, lactulose and polydextrose, exert beneficial effects in experimental and human intestinal inflammatory processes [7-13].

*Typha angustifolia* L. is a perennial aquatic macrophyta from the Typhaceae family that grows over broad climate and habitat ranges. This plant is named as taboa (Brazil), Lesser Bulrush (Britain) and narrow-leaved cattail or cattail (North America and other countries). *T. angustifolia* is characterised by its fast growth and high biomass [14]. Interestingly, several parts of the plant are edible, including dormant sprouts on the roots and bases of the leaves, ripe pollen, the stem and the starchy roots [15,16]. *T. angustifolia* and other species of the genus *Typha* are widely used as medicinal plants. In Brazil, Latin America and North America, the leaves are used as a diuretic, an astringent, a desiccant, a haemostatic agent and a vulnerary. In addition, the rhizomes are used as a diuretic, an astringent and an antimycobacterial. Moreover, the pollen is used in the treatment of scrofula, abscesses and abdominal pain, and the powder of the fuzz and rhizomes are used to prevent chafing, sores, inflammation, kidney stones and diarrhoea [17-20]. The rhizomes of *T. angustifolia* pods are also characterised by high fibre (17.20 g/100 g of flour) and carbohydrate (67.29 g/100 g flour) contents, and they are known to be rich in starch granules [16,21,22], which can be used by colonic microbiota as substrates for anaerobic fermentation and the production of SCFAs.

The current pharmacological treatments that are available for IBD include corticosteroids, aminosalicylates, immunosupressants and anti-TNF-α antibodies, but these pharmacological therapies are associated with serious side effects, particularly after long-term use. Glucocorticoids are widely used in the treatment of IBD in UC and CD patients, but glucocorticoids are not a long-term solution for anyone with IBD because they result in numerous unwanted side effects and can damage organ function years after ingestion, particularly if a high dose was administered for longer than 6 months [23]. Dissatisfaction with current conventional therapies has resulted in an increased use of complementary and alternative medicinal approaches, including dietary components with biological activity, such as fibre and other prebiotics and probiotics, which are currently estimated to be used in 49.5% of cases [24,25].

Given that cattail rhizome flour (*T. angustifolia* L.) is a medicinal plant used to treat inflammation and related disease and is an important source of dietary fibre and carbohydrates, the aim of the present study was to evaluate if dietary supplementation with cattail rhizome flour could act as a prebiotic and produce protective effects on the intestinal inflammatory process. In addition, we also investigated whether the combination of dietary supplementation with prednisolone, which causes serious side effects after long-term use, has synergistic effects.

## Methods

### Chemicals

All chemicals were obtained from Sigma (St. Louis, MO, USA).

### Plant material and diet preparation

The rhizomes of cattail (*T. angustifolia* L.) were collected in Botucatu city, São Paulo, Brazil, in September 2009. The plant material was authenticated by taxonomists from Irina Delanova Gemtchujnicov Herbarium (Institute of Biosciences, São Paulo State University, UNESP), where a voucher was deposited under numbers Botu 2767, Botu 2768, and Botu 2769.

After collection, the fresh cattail rhizomes were washed, chopped and dried at 50°C for 72 hr in a hothouse with forced air circulation and renewal. After drying, the rhizomes were powdered to produce flour. The final yield of cattail rhizome flour was 11% relative to the fresh weight. The flour was added at a ratio of 5%, 10% or 20% in previously sprayed animal feed (Labina-Purine). After homogenisation and pelletisation, the diet containing 5%, 10% and 20% of the cattail rhizome flour was obtained for use in the experiments. The ingredient composition of the diets is shown in Table 1.

**Table 1:** Ingredient composition of the diets fed to rats (g/100 g)

**Ingredients**	**Control diet**	**5% rhizome diet**	**10% rhizome diet**	**20% rhizome diet**
Protein mix	23.0	21.85	20.7	18.5
Mineral mix^1^	12.0	11.40	10.8	9.7
Fiber	5.0	4.75	4.5	3.6
Vitamin mix^2^	1.0	0.95	0.9	0.8
Fat	10.0	9.5	9.0	8.0
Fatty acids	5.5	5.22	4.95	4.4
Corn starch	32.0	30.40	28.8	25.7
Sugar mix	6.0	5.7	5.4	4.9
Soybean meal	2.5	2.38	2.25	2.0
Wheat bran	3.0	2.85	2.7	2.4
Cattail flour^3^	-	5.0	10.0	20.0

^1^ Mineral mixture provided the following amounts (mg/Kg): Mg, 1.7; Mn, 110.0; I, 1.0; Co, 2.0; Fe, 180.0; Zn, 110.0; Cu, 30.0, Se, 0.2, Na, 2.8, P, 8.5; Ca, 13.0.

^2^Vitamin mixture provided the following amounts (mg/Kg/diet): vitamin A (25600 UI); vitamin D3 (4000 UI); vitamin E (82 mg); vitamin K (6.4 mg), vitamin B12 (40 μg); vitamin B6 (11 mg), folic acid (13 mg); choline (2800 mg), biotin (0.16 mg), niacin (220 mg), thiamine (11 mg). pantothenic acid (90 mg)

^3^Rhizomes of cattail (*Typha angustifolia* L.) containing total fiber (17.25), protein (5.81%), lipids (0.90%), ash (8.80%) and non-nitrogenated coumpounds (67.29%) [15].

### Phytochemical analysis of cattail rhizome flour

The phytochemical profile of the cattail rhizome flour was determined according to previously described methods [26]. Briefly, a 70% methanol/water extract from cattail rhizome flour (300 g) was prepared by percolation. A portion of this extract (150.0 mL) was hydrolysed to produce a hydrolysed extract. In addition, an ether extract (300 g cattail rhizome flour) was prepared in a Soxhlet apparatus. The three extracts were submitted to phytochemical reactions for detection of the fixed acids, alkaloids, anthocyanins, anthocyanidins, aurones, antranols, quartenary bases, catechins, chalcones, cyanogenic heterosides, coumarins, flavones, flavonols, flavanones, flavononols, phenols, quinones, resins, saponins, steroids, triterpenoids and xanthones.

### Animals

Male Wistar rats (weighing 200–220 g) from the Central Animal House, São Paulo State University, UNESP, Botucatu city, SP, Brazil, were housed in standard environmental conditions (21°C, 60-70% humidity) under a 12-hour light/dark cycle and air filtration. The rats had free access to water and food (Labina-Purine, Brazil). The experimental protocol met the Guidelines of Animal Experimentation and was approved by the Commission of Ethics in Animal Experimentation (protocol number 042/04-CEAE), Institute of Biosciences, São Paulo State University–UNESP.

### Experimental design and induction of experimental colitis

The rats were randomly assigned into ten groups with eight animals in each. Two noncolitic groups were used: the first received normal diet and the second received an enriched diet that consisted of 20% of cattail rhizome flour for 21 days. The colitic rats were divided into eight groups. A TNBS control group received normal diet for 14 days prior to the induction of colitis and 7 days thereafter. Three colitic groups received an enriched diet with 5%, 10% or 20% of cattail rhizome flour in the same conditions as the TNBS control group. Three additional colitic groups received an enriched diet with 5%, 10% or 20% of cattail rhizome flour plus prednisolone orally administered at a dose of 2 mg/kg for 3 days prior to the induction of colitis and 7 days thereafter. To assess the effects of prednisolone alone, the remaining group received only prednisolone (2 mg/kg) for 3 days prior to the induction of colitis and 7 days thereafter. Prednisolone was administered by means of an oesophageal catheter (5 ml/kg). Rats from the noncolitic and the nontreated colitic groups were orally administered water. Colitis was induced using a previously described method [27]. After an overnight fast, the rats were anaesthetised with halothane and given 10 mg of TNBS dissolved in 0.25 ml of 50% (vol/vol) ethanol using a Teflon cannula (Dupont, Wilmington, DE, USA) inserted 6–8 cm into the anus. During and after TNBS administration, the rats were kept in a head-down position until they recovered from the anaesthesia. The rats in the noncolitic group received 0.25 ml of saline. The rats in all the groups were euthanised 7 days after the induction of colitis by an overdose of halothane.

### Assessment of colonic damage

Animal body weights, the occurrence of diarrhoea (as detected by perianal fur soiling), and total food intake for each group were recorded daily. Once the rats were killed, the colon was removed aseptically, placed on an ice-cold plate, longitudinally opened, and the luminal contents were collected for the microbiological studies (see below). After the tissue collection, the colonic segment was cleaned of fat and mesentery and blotted on filter paper. Each specimen was weighed, and its length was measured under a constant load (2 g). Using previously described criteria [28], the colon was scored for macroscopically visible damage on a 0–10 scale by two observers who were unaware of the treatment (no damage: 0; no ulcer, hyperaemia: 1; linear ulcer with no significant inflammation: 2; linear ulcer with inflammation at one site: 3; ≥ 2 sites of ulceration/inflammation: 4; ≥ 2 major sites of ulceration and inflammation or one site of ulceration/inflammation extending 1 cm along the length of the colon: 5; if damage covers 2 cm along the length of the colon, the score is increased by 1 for each additional centimetre of involvement: 6 to 10). The colon was divided into different longitudinal pieces to be used for the following biochemical determinations: myeloperoxidase (MPO) activity, alkaline phosphatase (AP) activity and total glutathione (GSH) content.

### Microscopic assessment of colitis

Prior to the collection of longitudinal pieces for biochemical analysis, representative transversal whole-gut specimens were taken from a region of the inflamed colon that corresponded to the segment adjacent to the gross macroscopic damage, and the specimens were fixed in 4% buffered formaldehyde. Cross-sections were selected and embedded in paraffin. Equivalent colonic segments were also obtained from the noncolitic group. Full thickness sections of 5 mm were obtained at different levels and were stained with haematoxylin and eosin. The histological damage was evaluated by one observer who was blinded to the experimental groups as previously described [29].

### Biochemical evaluation

MPO activity was determined according to the technique described by Krawisz et al. [30]. The results are expressed as MPO units/g of tissue. One unit of MPO activity was defined as the amount required to degrade 1 mmol of hydrogen peroxide/min at 25°C.

AP activity was determined spectrophotometrically using disodium nitrophenylphosphate (5.5 mM) as the substrate in 50 mM glycine buffer with 0.5 mM MgCl_2_ at pH 10.5 [31]. The enzymatic activity is expressed as mU/mg of protein [32].

GSH content was quantified with the recycling assay as described by Anderson et al. [33], and the results are expressed as nmol/g of wet tissue.

### Antioxidant activity

Additional *in vitro* experiments were performed to test the antioxidant activity of different concentrations of cattail rhizome flour (2.5–1400 μg/ml). The antioxidant activity was evaluated by inhibition of induced lipid peroxidation in rat brain membrane as previously described [34]. The flavonoid quercetin was used as a reference and tested in the same assay system.

### Microbiological studies

Luminal content samples were weighed, homogenised, and serially diluted in sterile 0.85% saline. Serial 10-fold dilutions of homogenates were plated on Man Rogosa and Sharp Agar (MRS), a specific medium for lactic acid bacteria, and incubated under microaerobic conditions (5% CO_2_) at 35°C for 120 hr. After the incubation, the final count of colonies was reported as log10 colony-forming units per gram of faecal material.

### Statistical analysis

All results are expressed as mean ± SEM, and differences between the means were tested for statistical significance using one-way analysis of variance and post hoc least significance tests. Nonparametric data (scores) are expressed as the median (range) and were analysed with a Kruskal-Wallis test. Differences between proportions were analysed with a χ^2^ test. Statistical significance was set at *p* < 0.05.

## Results

### Macroscopic, biochemical and histological evaluation

**Figure 1:**
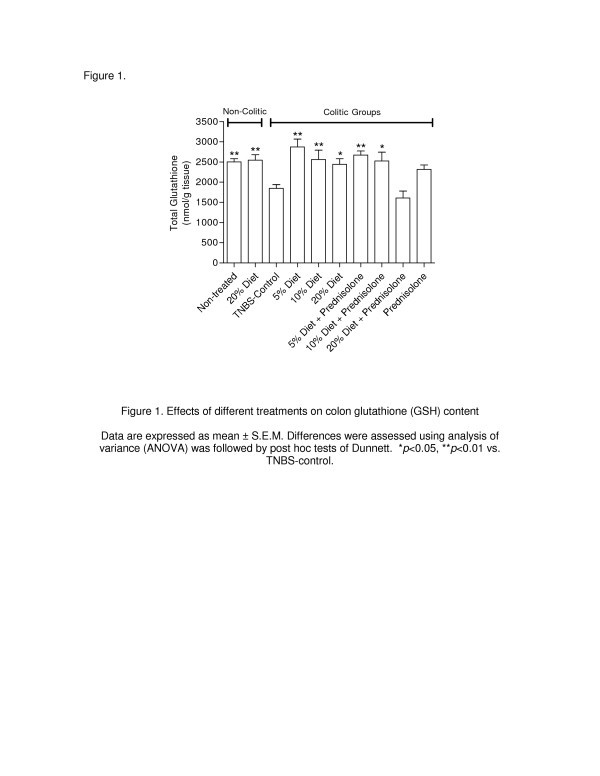
**Photomicrography of colon of different groups: (A)** Non-colitic group; **(B)** TNBS-control group; **(C)** prednisolone (2 mg/kg); **(D)** 5% cattail rhizome flour; **(E)** 10% cattail rhizome flour; **(F)** 5% cattail rhizome flour + prednisolone (2 mg/kg); **(G)** 10% cattail rhizome flour + prednisolone (2 mg/kg).

**Figure 2:**
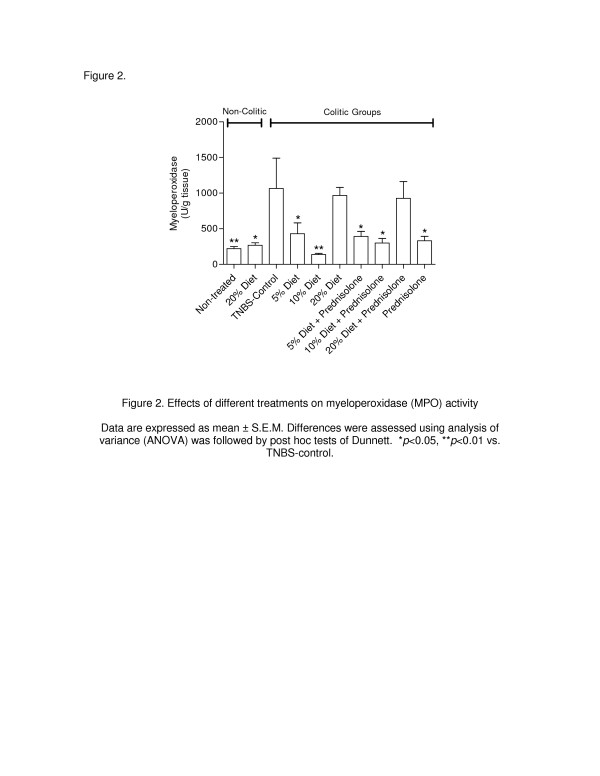
**Effects of different treatments on colon glutathione (GSH) content.** Data are expressed as mean ± S.E.M. Differences were assessed using analysis of variance (ANOVA) was followed by post hoc tests of Dunnett. *p < 0.05, **p < 0.01 vs. TNBS-control.

**Figure 3:**
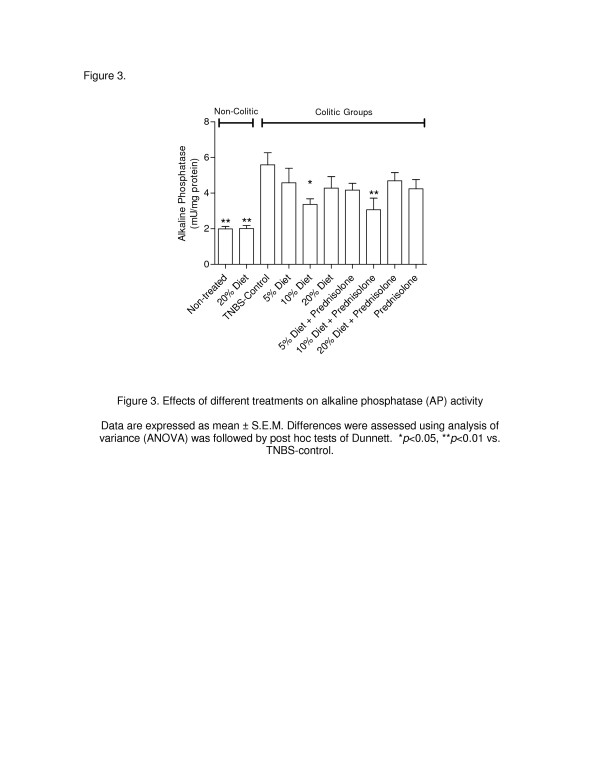
**Effects of different treatments on myeloperoxidase (MPO) activity.** Data are expressed as mean ± S.E.M. Differences were assessed using analysis of variance (ANOVA) was followed by post hoc tests of Dunnett. *p < 0.05, **p < 0.01 vs. TNBS-control.

**Figure 4:**
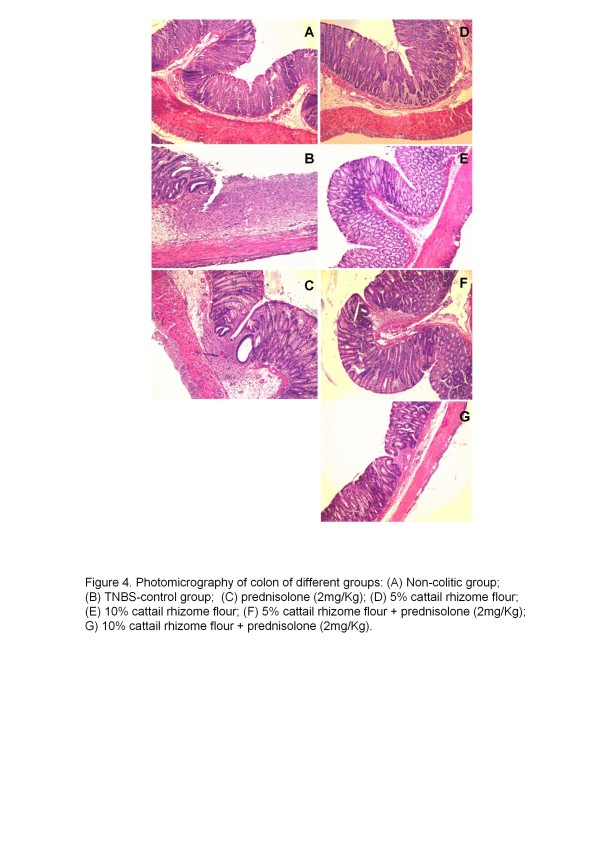
**Effects of different treatments on alkaline phosphatase (AP) activity.** Data are expressed as mean ± S.E.M. Differences were assessed using analysis of variance (ANOVA) was followed by post hoc tests of Dunnett. *p < 0.05, **p < 0.01 vs. TNBS-control.

TNBS instillation resulted in colonic inflammation, which was evident after 7 days by severe necrosis of the mucosa (typically extending 2.71–3.37 cm along the colon), bowel wall thickening, and hyperaemia (Table 2). TNBS-induced colon inflammation was associated with a significant increase in the colonic weight/length ratio, the incidence of adherence to adjacent organs (Table 2, *p* < 0.01), signs of diarrhoea in 100% of the colitic animals and a reduction in food intake compared with the noncolitic rats (data not shown). Histological assessment of the colonic samples from the TNBS control group revealed severe transmural disruption of the normal architecture of the colon, extensive ulceration, inflammation involving all the intestinal layers of the colon, and a marked neutrophill infiltration (Figure 1B). The colonic samples from the TNBS control group received a microscopic score of 10.5 (Table 2), which differed significantly from that for the noncolitic groups (*p* < 0.01). The colonic damage was biochemically characterised by a significant reduction (*p* < 0.01) in colonic GSH levels (Figure 2), a 3.8-fold increase in MPO activity (*p* < 0.01) and a 2.8-fold increase (*p* < 0.01) in AP activity (Figures 3 and 4) as compared with the noncolitic rats.

**Table 2:** Effects of different treatments on damage score, extension of lesion, changes in colon weight, adherences to adjacent organs and microscopic score

**Experimental Groups**	**Score**^**a**^**(0–10)**	**Extension of lesion**^**b**^**(cm)**	**Colon weight**^**b**^**(mg/cm)**	**Adherence(%)**	**Microscopic Score (0-27)**^**a**^
Non-colitic groups					
Non treated	0***	0**	112.68 ± 6.28**	0**	0**
20% diet	0***	0**	113.63 ± 5.56**	0*	0**
Colitic groups					
TNBS-Control	7 (4–8)	3.04 ± 0.33	260.63 ± 37.62	60	10.5 (5–19)
5% diet	6 (0–7)	1.67 ± 0.42*	156.67 ± 16.76*	15*	8 (5–15)
10% diet	5 (4–6)	1.45 ± 0.29*	169.24 ± 6.12*	0*	5 (3–11)
20% diet	6 (4–8)	2.17 ± 0.36	204.75 ± 22.81	85	4 (2–10)
5% diet + prednisolone	7 (5–10)	3.24 ± 0.59	196.73 ± 23.22	15*	5 (2–14)
10% diet + prednisolone	6 (5–7)	2.43 ± .029	156.01 ± 9.87*	45	7 (2–11)
20% diet + prednisolone	6 (0–8)	2.41 ± 0.68	258.04 ± 63.77	45	5 (1–9)
prednisolone	3 (0–4)*	1.40 ± 0.27*	162.20 ± 8.14*	45	11.0 (8–14)

^a^ Score and microscopic score data are expressed as median (range) and were statically evaluated by non-parametric Kruskal-Wallis test; ^b^ Extension of lesion and colonic weight data are expressed as mean ± SEM values and differences were assessed using ANOVA was followed by post hoc test of Dunnet; Adherence was expressed in percentage and was analyzed with the χ^2^ test. *P < 0.05, P < 0.01 and *P < 0.001* versus TNBS control group.

The treatment of noncolitic rats with the 20% cattail rhizome diet for 21 days had no effect on the colon weight and there were no signs of diarrhoea or adherence to adjacent organs. In addition, we observed no effect on GSH levels or MPO and AP activities, and all the results in the noncolitic rats that received the 20% cattail rhizome diet were similar to those for the untreated noncolitic group (Table 2, Figures 2– 4). Interestingly, the dietary treatment of the colitic rats for 14 days prior to the induction of colitis and 7 days thereafter showed an overall lower impact of TNBS-induced colonic damage compared with the TNBS control group.

The colitic rats treated with 5% and 10% cattail rhizome flour showed a faster weight gain in comparison with the weight loss observed in the rats from the TNBS control group (7.5 ± 0.92 weight gain in the treated rats *vs.* 12.5 ± 3.1% weight loss in TNBS control rats), but no effect on weight gain was observed in the rats that were treated with 20% cattail rhizome diet (12.5 ± 3.1% weight loss in the TNBS control rats *vs.* 1.42 ± 0.62 weight gain in the treated rats). Feeding a 5% or 10% cattail rhizome diet to the colitic rats resulted in a significant reduction in the extension of the lesions (1.67 ± 0.42 and 1.45 ± 0.29, respectively, in the treated rats *vs.* 3.04 ± 0.33 in the TNBS controls, *p* < 0.05), the weight/length ratio (156.67 ± 16.76 and 169.24 ± 6.12, respectively, in the treated rats *vs.* 260.63 ± 37.62 in the TNBS controls, *p* < 0.05) and the incidence of adherence to adjacent organs (15% and 0%, respectively, in the treated rats *vs.* 60% in the TNBS controls, *p* < 0.05) (Table 2). Interestingly, the dietary intervention with 20% cattail rhizome flour did not produce any effects on these parameters. No effects were observed in the microscopic scores after all the cattail rhizome treatments, but the histological studies showed that the flour treatment was able to ameliorate some of the processes involved in colon inflammation (primarily leukocyte infiltration) compared with the TNBS controls (Figure 1D and 1E). The intestinal anti-inflammatory effect was also observed biochemically by significant counteractions of the colonic GSH level compared with the TNBS control group (Figure 2, *p* < 0.01). In the rats treated with 5% and 10% cattail rhizome flour, the observed decrease in leukocyte infiltration observed with histological studies was confirmed by a significant reduction in MPO activity (Figure 3, *p* < 0.05 and *p* < 0.01for the 5% and 10% cattail flour, respectively). In addition, AP activity was significantly reduced (*p* < 0.05) in the rats fed the 10% dietary cattail rhizome diet, whereas AP activity was increased in the TNBS control group (Figure 4).

When colitic rats were treated with a combination of the cattail rhizome diet and prednisolone, protective effects were observed in the rats that received the 10% cattail rhizome flour; however, the combination of prednisolone and the diet containing 5% or 20% cattail rhizome flour showed no effect on the macroscopic and microscopic scores, the extension of the lesion, the colon weight ratio or the adherence to adjacent organs (Table 2). The combination of prednisolone and the diets containing 5% or 10% cattail rhizome flour displayed a significant beneficial effect in the colitic rats, as evidenced by an attenuation of the depletion of GSH (Figure 2, *p* < 0.01) and a reduction of MPO activity (Figure 3, *p* < 0.05 and *p* < 0.01 for 5% and 10% cattail flour, respectively). Interestingly, only the combination of the 10% cattail rhizome flour and prednisolone was able to significantly inhibit AP activity (Figure 4, *p* < 0.05). This effect was also confirmed by histological studies that showed a pronounced recovery of colon cytoarchitecture accompanied by a mild ulceration in mucosa and a reduction of the inflammatory cells in the submucosa (Figure 1F and 1G).

Prednisolone, which was used as a reference drug, showed anti-inflammatory effects as evidenced by significant reductions in the macroscopic score, the extension of the lesion and the colon weight ratio (Table 2, *p* < 0.05). The protective effects of prednisolone were also biochemically related to a reduction of MPO activity (Figure 3, *p* < 0.05). Histological analysis also indicated a protective effect (Figure 1C). Prednisolone had no effect on the microscopic scores, the adherences to adjacent organs (Table 2), the GSH level or AP activity (Figure 4).

### Microbiological analysis

No significant difference in the lactic acid bacteria counts was found between the normal and colitic rats. The diets that contained 5%, 10% or 20% cattail rhizome flour did not promote any alterations in the lactic acid bacteria counts in the noncolitic or the colitic rats (data not shown).

### Anti-oxidant activity

The *in vitro* antioxidant experiments demonstrated that cattail rhizome flour exerts a concentration-dependent inhibitory effect on the lipid peroxidation induced in rat brain membranes, with an IC_50_ value of 116.14 ± 4.91 μg/ml. The corresponding IC_50_ value of quercetin was 0.39 ± 0.02 μg/ml.

### Qualitative phytochemical analysis

The qualitative phytochemical analysis indicated the presence of saponins, flavonoids and coumarin derivatives in the cattail rhizome flour.

## Discussion

Current pharmacological treatments for IBD include a series of compounds, such as aminosalicylates, corticosteroids, biological agents and immunosuppressants, but the long-term use of these drugs results in unwanted and serious side effects. Therefore, a combination of products that improve the anti-inflammatory activity of drugs would be an important approach for IBD treatment. The present study was designed to evaluate novel experimental interventions using cattail rhizome flour as a potential dietary product and complementary agent to be used in association with prednisolone, which is used for treatment of human IBD.

Initially, we evaluated the effects of enriched diet with 20% cattail rhizome flour in healthy rats. After three weeks, we did not observe any diet-induced changes in food intake or in clinical, macroscopic, microscopic or biochemical parameters (data not shown), which suggested that cattail rhizome flour is a safe product for use in the diet and is palatable to rats.

In another set of experiments, we evaluated the effects of 5%, 10% and 20% cattail rhizome flour on the intestinal inflammatory process induced by TNBS in rats. Our data demonstrate that 5% and 10% cattail rhizome diets prevented intestinal inflammatory processes, whereas 20% cattail rhizome flour only prevented GSH depletion induced by TNBS. The protective effects produced by dietary treatment with 5% and 10% cattail rhizome flour were similar to those produced by prednisolone.

We also evaluated the effects of cattail rhizome flour on the intestinal anti-inflammatory activity of prednisolone to investigate whether cattail rhizome flour improves the pharmacological activity of this currently used glucocorticoid. Our results revealed that the combination of prednisolone (2 mg/kg) and the 10% cattail rhizome diet was effective in preventing the intestinal inflammatory process induced by TNBS in rats. Interestingly, there was no evidence of synergistic effects for this combination treatment.

In all of the experiments, we evaluated the effect exerted by the cattail rhizome flour diet on the growth and development of lactic bacteria, and we demonstrated that protective effects on the colon damage induced by TNBS were not related to increased lactic bacteria counts. However, the methods used to determine bacteria growth and development are limited, and new studies are necessary, particularly studies that use more appropriate and specific culture medium and evaluation of other bacteria with prebiotic effects (e.g., bifidobacteria).

The detected intestinal anti-inflammatory activity of *Typha angustifolia* rhizomes correlates with traditional uses against inflammation and diarrhoea [7-10]. Few pharmacological studies with *Typha angustifolia* have been performed, but its effects as an immunoregulating product can corroborate the anti-inflammatory activity that was demonstrated in the present study [35]. This plant has also been described as a potential anti-aging and antidiabetic agent and has been shown to improve metabolism and reduce aldose reductase in streptozotocin-induced diabetic mice [35,36]. Several plants of the genus *Typha* have been proved to exert anti-inflammatory effects related to inhibition of neutrophil functions; inhibition of nitric oxide, interleukin-4, interleukin-5, interleukin 13 and prostaglandin production; and inhibition of the expression of inducible nitric oxide synthase and cyclo-oxygenase 2 [37-39].

Cattail rhizome flour attenuated the GSH depletion induced by colonic inflammatory processes and restored the levels toward the normal value. GSH depletion after intestinal damage in rats is a typical characteristic of colitis induced by TNBS [11,12,34,40,41]. GSH plays a key role in controlling the redox state of the cell by acting as a scavenger of reactive oxygen species and keeping the enzyme GSH peroxidase in a reduced state [42]. Indeed, studies have reported that GSH supplementation improves oxidative damage in TNBS colitis [43]. Thus, the use of antioxidant compounds may be useful in limiting damage in IBD. Interestingly, studies have proposed that antioxidant activity may be partially responsible for the beneficial effects of glucocorticoids (primarily prednisolone), such as the inhibition of nitric oxide production in intestinal inflammation [44]. Indeed, previous studies have shown the beneficial effects of different antioxidant compounds in experimental models of rat colitis, including flavonoids [45] vitamin E [46], tempol [47], and coumarin derivatives [34,40,41]. In addition, the antioxidant effect of cattail rhizome flour was confirmed by an *in vitro* lipid peroxidation assay. This activity was lower than the effects of quercetin but comparable to the standardised plant extracts of *Vochysia tucanorum, Baccharis dracunculifolia, Coccoloba uvifera* and *Cissus cyssoides*[48-51].

The intestinal anti-inflammatory activity of the cattail rhizome flour supplementation was also related to an inhibitory effect on MPO activity. MPO, an enzyme found predominantly in the azurophilic granules of neutrophils, is a biochemical marker of neutrophil infiltration, and measurements of its activity have been widely used to detect intestinal inflammatory processes [52,53]. Reduction of MPO activity can be interpreted as a manifestation of the anti-inflammatory property of a given compound [54]. Colonic tissue MPO activity, which was markedly elevated in the TNBS control rats, was significantly decreased in the rats treated with dietary cattail rhizome flour, and this effect was supported by the number of neutrophils detected in the tissues by histological analysis.

Human IBD is characterised by an increased expression of markers of differentiation and AP, which is upregulated in experimental chronic diarrhoea, has been considered a phenotypic marker of differentiation, [55]. Moreover, colonic tissue AP activity has been used to differentiate Crohn’s disease from ulcerative colitis because AP enzymatic activity is higher in Crohn’s disease [56]. A recent study showed that intestinal AP mRNA expression is reduced in patients with UC and CD and that oral administration of active intestinal AP enzymes in the rat intestinal tract results in a significant reduction of inflammation [57]. In the present study, colon AP activity in colitic rats was significantly increased as compared with the noncolitic rats. After treatment with 10% cattail rhizome flour or the combination of 10% cattail rhizome flour and prednisolone, an inhibitory effect on the AP activity was detected. Because prednisolone was ineffective in inhibiting this enzymatic activity by itself, it is possible that the inhibitory effect produced by the combination of cattail and prednisolone is related to the use of 10% cattail rhizome flour.

Finally, our qualitative phytochemical studies revealed the presence of flavonoids, coumarins and saponins in cattail rhizome flour. Various antioxidant flavonoids and coumarin derivatives have been reported as protective products to prevent and treat intestinal inflammatory processes induced by different chemical inductors of experimental colitis [34,40,41,45]. Several studies have shown that different saponins from *Panax ginseng* and *Codonopsis lanceolata* were active compounds in experimental colitis [58-60]. Hence, it is plausible that the presence of these classes of natural compounds in the rhizome flour of cattail contribute to the observed intestinal anti-inflammatory activity.

## Conclusions

Based on our results, we conclude that dietary supplementation with 10% cattail rhizome flour and its combination with prednisolone prevent TNBS-induced colonic damage in rats. This effect was associated with an improvement in intestinal oxidative stress, probably derived from antioxidant properties of active compounds detected in the cattail rhizome, especially flavonoids and coumarins. Indeed, the observed protective effect was not related to an improvement in lactic bacteria counts. The present results suggest that cattail rhizome flour constitutes an important dietary supplement and complementary medicinal product for the prevention and treatment of human IBD. Because of limitations of the study, however, further studies are necessary to gain more knowledge about the intestinal anti-inflammatory properties and the chemical composition of cattail rhizome flour.

## Competing interests

The authors declare that they have no financial or nonfinancial competing interests.

## Authors’ contributions

ACF and LNS carried out the study, data collection and analysis. VLMR supervised the microbiological studies and their data analysis. LCDS designed the study and supervised the inflammatory bioassays, biochemical evaluation, phytochemical studies and data analysis. ACF and LCDS prepared the draft of the manuscript. All the authors read and approved the final manuscript.

## Pre-publication history

The pre-publication history for this paper can be accessed here:

http://www.biomedcentral.com/1472-6882/12/62/prepub
